# Safety and effect of high dose allopurinol in patients with severe left ventricular systolic dysfunction

**DOI:** 10.15171/jcvtr.2017.17

**Published:** 2017-05-17

**Authors:** Mohammad Mostafa Ansari-Ramandi, Majid Maleki, Azin Alizadehasl, Ahmad Amin, Sepideh Taghavi, Mohammad Javad Alemzadeh-Ansari, Ali Kazem Moussavi, Nasim Naderi

**Affiliations:** Rajaie Cardiovascular Medical and Research Center, Iran University of Medical Sciences, Tehran, Iran

**Keywords:** Allopurinol, Severe LV Systolic Dysfunction, Strain Imaging, Quality of Life, NT-proBNP

## Abstract

***Introduction:*** Allopurinol used in the treatment of gout has been shown to improve the vascular endothelial dysfunction and reduce the dysfunction of the failing heart. This study was done to evaluate the effect and safety of allopurinol in non-hyperuricemic patients with chronic severe left ventricular (LV) dysfunction.

***Methods:*** In this study, 35 consecutive cases of non-hyperuricemic patients with chronic heart failure who had severe LV systolic dysfunction (ejection fraction of less than 35%) and were on optimal guideline directed medical therapies for at least 3 months were included. Allopurinol was administered with the dose of 300 mg po daily for 1 week and then it was up-titrated to a dose of 600 mg po daily for 3 months. Six minute walk test, strain imaging, laboratory testing were done for every patient at baseline and after 3 months treatment with allopurinol.

***Results:*** In this study 30 heart failure (HF) patients with a mean age of 49.3 ± 14.4 years old were evaluated. No adverse effects were reported except for one case of skin rash after 4 days treatment which was excluded from the study. Study showed significant improvement of six minute walk test of the patients from 384.5 ± 81.5 meters to 402.8 ± 89.6 meters and the global longitudinal peak strain (*P *< 0.001). There was also significant decrease in the level of erythrocyte sedimentation rate and N-terminal pro-brain natriuretic peptide (NT-proBNP) after 3 months.

***Conclusion:*** Allopurinol could be of benefit in non-hyperuricemic patients with severe LV systolic dysfunction without significant adverse effects. Randomized clinical trials are needed in future to confirm the results.

## Introduction

Heart failure (HF) is a systemic syndrome leading to hemodynamic failure and neuroendocrine activation.^1^ The HF syndrome is progressive with high morbidity and mortality. Different mechanisms have been identified to contribute to the development of HF including endothelial dysfunction, oxidative stress and inflammatory factors.^1,2^ Some studies have shown increased activation and expression of xanthine oxidase (XO) in failing myocardial tissue.^2^ XO catalyzes the production of large amounts of reactive oxygen species (ROS) which have been found to mediate myocardial dilation and hypertrophy and to increase endothelial dysfunction. Allopurinol which is a XO inhibitor and used in the treatment of gout has been shown to improve the vascular endothelial dysfunction and reduce the dysfunction of the failing heart. Allopurinol use reduces oxygen free radicals formation as well as oxidative damage and has favorable effects on myocardial energy metabolism, endothelial function, mechanoenergetic coupling, myocardial oxygen consumption, ventricular remodeling and clinical status in the setting of HF.^3-9^ However, allopurinol’s effect is under question in HF patients without hyperuricemia.^2-13^

In the present study we aimed to assess the effects of allopurinol and its tolerability in terms of presence of side effects in HF patients without hyperuricemia.

## Patients and Methods

### Patient selection

Among patients referred to outpatient HF clinic, 35 HF patients were consecutively enrolled from February 2016 to March 2016 according to the following inclusion/exclusion criteria. Symptomatic or asymptomatic HF patients (New York Heart Association class of I-III) with severe left ventricular (LV) systolic dysfunction (ejection fraction ≤35%, HFrEF), absence of hyperuricemia (uric acid less than 6 mg/dL and 6.8 mg/dL in women and men respectively), age ≥18 years and participant willingness to be enrolled into the study. All study participants had to be on guideline-directed medical therapy for at least 3 months before enrollment.

The patients were excluded if they were older than 80 years or had one of the following conditions; significant renal insufficiency (glomerular filtration rate of less than 60 mL/min or serum creatinine level of more than 3 mg/dL), severe hepatic failure; use of azathioprine, 6-mercaptopurin and warfarin as well as known history of sensitivity to allopurinol. Patients were also excluded if they developed decompensation or had any change in their HF medications during follow up.

### Data acquisition and laboratory measurements

A thorough clinical history was obtained and a comprehensive physical examination was performed for all study participants.

The New York Heart Association (NYHA) function class was assessed considering the severity of the limitations in physical activities, where class I indicates no symptom (dyspnea), class II indicates presence of symptoms in ordinary activities, class III indicates symptoms at less than ordinary activities and finally class IV indicates symptoms of dyspnea at rest.

### Physical performance assessment

The physical performance and exercise tolerance of patients were assessed by 6-minute walk test (6MWT) according to the standard protocol.^14^

### Biochemical measurements

Biochemical measurements were performed at baseline and after 3 months treatment with allopurinol .Whole blood was collected from all study participants after 12 hours overnight fasting. The laboratory tests including complete blood count (CBC), hemoglobin level, erythrocyte sedimentation rate (ESR), blood urea nitrogen(BUN),creatinine, serum electrolytes (sodium, potassium, magnesium) liver function tests (alanine aminotransferase [ALT], aspartate aminotransferase [AST], alkaline phosphatase [ALKP], total and direct bilirubin levels), N- terminal pro-brain natriuretic peptide (pro –BNP) and high sensitivity C-reactive protein (hs-CRP) were performed at our laboratory on the day of blood collection

### Echocardiographic examination

Two-dimensional (2D) conventional, pulse, and transthoracic echocardiographic study was performed with commercial GE Vivid 7 System (Horten, Norway), equipped with an M3S multi-frequency harmonic phased array transducer for the assessment of strain imaging parameters via the tissue Doppler-based method. Images were acquired from the subjects at rest, lying in the left lateral supine position at the end of expiration. Two-dimensional Electrocardiography (ECG) was superimposed on the images, and end-diastole was considered at the peak R-wave of the ECG. The LV global systolic function was evaluated using a modified biplane Simpson method for calculating the LV ejection fraction (LVEF) by measuring end-diastolic and end-systolic volumes in the 2D images.

The right ventricle (RV) systolic function was evaluated using the visual assessment and following parameters: tissue Doppler-derived tricuspid lateral annular systolic velocity (Sm) and tricuspid annular plane systolic excursion (TAPSE).

However the concept of strain is complex.

ε=L−L0/L0=ΔL/L0

Where ε = strain, L0 = baseline length and L = instantaneous lengths at the time of measurement.

The amount of deformation (positive or negative strain) is usually expressed in %. Positive strain values describe thickening, negative values describe shortening, of a given myocardial segment related to its original length. During myocardial contraction, as the wall shortens it also thickens and thus assessment of all parameters, radial thickening (positive strain), circumferential shortening (negative strain) and longitudinal shortening (negative strain), is useful for the evaluation of contractile function.

### Assessment of quality of life

Patients were also asked to complete the Iranian questionnaire to assess quality of life in patients (IHF-Qol) with HF at baseline and after three months treatment with allopurinol.

IHF-Qol is the first Iranian questionnaire to assess quality of life in patients with HF which has been developed and validated by a study done by Naderi et al^15^ It comprises of 5 different domains of quality of life which include 15 questions and a final conclusive question. The domains evaluated are symptoms and their severity, physical limitations, social interference, psychological condition and self-efficacy and knowledge. A score equal to 32–47 was considered as moderate satisfaction and the more the score was better satisfaction was concluded.

### Allopurinol administration

Allopurinol was administered with the dose of 300 mg po daily for a week and after being sure of not having sensitivity to allopurinol it was up titrated to a dose of 600 mg po daily for 3 months.

Patients had complete access to their physician to report any side effects while taking the medication and could have early examination before 3 months if needed.

The patients were evaluated and assessed after 3 months for any side effects and 6-minute walk test, echocardiography, strain imaging and laboratory testing were repeated for the patients.

### Statistical analysis

Data are presented as frequencies, mean ± standard deviation (SD) or median ± interquartile range (IQR) as appropriate. For statistical analysis one sample Kolmogorov-Smirnov test was used to assess the normal distribution of variables and then paired sample *t* test was used for parametric numeric data and Wilcoxon for non-parametric numeric data. Mcnemar test was used for nominal data. *P* value of less than 0.05 was regarded as significant. All analyses were done using IBM SPSS statistics 19 for Windows (IBM Corp, Armonk, NY, USA).

## Results

### Clinical data

Of the original recruited patients five did not complete the study. During the study period we had one missed patient, one patient showed transient mild skin rash so allopurinol was stopped, two developed decompensated HF due to poor drug compliance and another patient developed acute renal failure.

The clinical and demographic data of thirty patients at baseline are presented in Table 1. Table 2 is a comparison between the clinical data of patients at baseline and after 3 months treatment with allopurinol. The mean age of the patients was 49.37 ± 14.44 years and 46.7% of the patients were male. All patients were on standard recommended HF medications including angiotensin converting enzyme inhibitor (ACEI)/angiotensin receptor blocker (ARB) [100%], beta blocker (100%), mineralocorticoid receptor antagonist (MRA) [100%] and diuretics (100%).

**Table 1:** Demographic and clinical data of the patients at baseline

**Index**	**At baseline**
Age, years, mean ± SD	49.3±14.4
Gender, N (%)	
Male	14 (46.7)
Female	16 (53.3)
Cause of cardiomyopathy, N (%)	
Ischemic	15 (50)
Dilated	15 (50)
Hypertension, N (%)	9 (33.3)
Dyslipidemia, N (%)	12 (40)
Diabetes mellitus, N (%)	4 (13.3)
Smoking, N (%)	11 (36.7)
ICD, N (%)	8 (26.7)

Abbreviation: ICD, Implantable cardioverter defibrillator.

**Table 2:** Comparison between clinical data of patients at baseline and after three months treatment with allopurinol

** Index**	**At baseline**	**After treatment**	***P***
EF, %, mean (SD)	21.50 (6.8)	22.16 (7.6)	0.35
NYHA FC, N (%)			
FC I	14 (46.7)	28 (93.3)	<0.001
FC II	10 (33.3)	2 (6.7)	
FC III	6 (20)	0 (0)	
Orthopnea, N (%)	14 (46.7)	7 (23.3)	0.016
PND, N (%)	8 (26.6)	6 (20)	0.5
Swelling, N (%)	9 (30)	7 (23.3)	0.5
Fatigue, N (%)	12 (40)	6 (20)	0.03
Anorexia, N (%)	9 (30)	4 (13.3)	0.06
Low back pain, N (%)	9 (30)	8 (26.6)	1
Leg pain, N (%)	9 (30)	4 (13.3)	0.06
Muscle cramp, N (%)	9 (30)	3 (10)	0.03
Nausea and vomiting, N (%)	3 (10)	2 (6.7)	1
Abdominal pain, N (%)	1 (3.3)	1 (3.3)	1
Skin rash, N (%)	1 (3.3)	2 (6.7)	1

Abbreviations: EF, ejection fraction; NYHA FC, New York Heart Association Functional Class; PND, paroxysmal nocturnal dyspnea.

### Patients’ clinical status and exercise capacity at baseline and after 3 months treatment with allopurinol

The most common symptom of patients at baseline was orthopnea (46.7%). However, as shown in Table 2, after 3 month treatment with allopurinol the patients were improved significantly in terms of symptoms particularly dyspnea .

The 6-minute walk test was significantly improved after 3 months treatment with allopurinol (384.5±81.52 meters at base line versus 402.83±89.61 after three months, *P*<0.001)

### Biomarkers and laboratory tests

As was expected the serum uric acid level decreased significantly after 3 months (5.9 [1.3] mg/dL at base line versus 3.1 [0.9] mg/dL after 3 months, *P* < 0.001) .The NT-pro BNP level was significantly decreased following treatment with allopurinol (782 [522.7-1000] ng/mL at baseline versus 630 [405-895.7] ng/mL after 3 months, *P* < 0.001). There was also significant reduction in erythrocyte sedimentation rate (ESR). However, as shown in Table 3, no other remarkable changes were observed in terms of kidney function, electrolytes and liver function tests.

**Table 3:** Changes in laboratory test results of patients after three months of treatment with allopurinol

**Index**	**At baseline**	**After treatment**	***P*** ** value**
BUN, mg/dL, mean (SD)	21.2 (6.6)	22.6 (12.0)	0.33
Cr, mg/dL, mean (SD)	0.9 (0.2)	0.9 (0.2)	0.84
Hb, g/dl, mean (SD)	13.3 (1.9)	13.2 (1.8)	0.82
Mg, mg/dL, mean (SD)	1.9 (0.1)	2.1 (0.1)	0.07
K, mg/dL, mean (SD)	4.2 (0.4)	4.1 (0.5)	0.29
Uric acid, mg/dL, mean (SD)	5.9 (1.3)	3.1 (0.9)	<0.001
AST, units/L, mean (SD)	20.9 (6.3)	20.8 (6.7)	0.81
ALT, units/L, median (IQR)	21 (17-24)	20 (17-24)	0.51
ALP, units/L, mean (SD)	219.2 (68.2)	226.1 (68.8)	0.70
Total bilirubin, mg/dL, median (IQR)	0.9 (0.5-1.1)	0.9 (0.5-1.1)	0.59
Direct bilirubin, mg/dL, mean (SD)	0.3 (0.1)	0.2 (0.1)	0.14
ESR, mm/h, mean (SD)	24 (17.6)	16 (12.3)	<0.001
hs-CRP, mg/L, median (IQR)	4.8 (4.7-5.2)	5 (4.1-5.4)	0.71
NT-pro BNP, ng/mL, median (IQR)	782 (522.7-1000)	630 (405-895.7)	<0.001

Abbreviations; BUN, blood urea nitrogen; Cr, creatinine; Hb, hemoglobin; Mg, magnesium; K, potassium; AST, aspartate aminotransferase; ALT, alanine aminotransferase; ALP, alkaline phosphatase; ESR: erythrocyte sedimentation rate; hs-CRP: high sensitivity C reactive protein; NT-pro BNP, N terminal pro brain type natriuretic peptide; SD, standard deviation; IQR, interquartile range.

### Echocardiography and strain imaging measures

There was no significant improvement of the global LVEF and the RV function after 3 months. However, the global longitudinal peak strain in all different views and in average was improved significantly (Table 4).

**Table 4:** Changes in strain imaging in patients after three months treatment with allopurinol

**Index**	**At baseline**	**After treatment**	**P value**
GLPS Avg, %, mean (SD)	-9.3 (3.2)	-10.9 (4.4)	<0.001
GLPS LAX, %, mean (SD)	-9.4 (4.3)	-10.2 (4.9)	<0.001
GLPS A4C, %, mean (SD)	-9.7 (3.8)	-10.9 (4.4)	<0.001
GLPS A2C, %, mean (SD)	-9.1 (2.9)	-11.5 (3.9)	<0.001
			

Abbreviations: GLPS Avg, average global longitudinal peak strain; GLPS LAX, apical long axis view global longitudinal peak strain; GLPS A4C, four chamber view global longitudinal peak strain; GLPS A2C, two chamber view global longitudinal peak strain; SD, standard deviation.

### Changes in IHF-QoL score

The data of the IHF-QoL scores of the patients are shown in Table 5 which shows a significant improvement in the score after three months in the total score and the 5 domains of symptoms and severity, physical limitations, social interference, psychological condition and self-efficacy and knowledge. The conclusive score was also improved significantly after 3 months.

**Table 5:** Changes in IHF-Qol score of patients after three months treatment with allopurinol

**Index**	**At baseline**	**After treatment**	***P*** ** value**
IHF Qol total score, mean (SD)	52.0 (6.4)	57.6 (7.8)	<0.001
IHF Qol symptoms score, mean (SD)	15.6 (2.5)	16.8 (2.5)	<0.001
IHF Qol physical limitations score, mean (SD)	12.3 (2.3)	13.5 (2.6)	<0.001
Social interference score, mean (SD)	9.7 (1.2)	10.7 (1.4)	<0.001
Psychological condition score, mean (SD)	7.6 (1.6)	8.8 (1.9)	<0.001
Self-efficacy and knowledge score, median (IQR)	4 (3-6)	5.5(4.7-6)	0.001
Conclusive score, median (IQR)	2 (2-3)	3 (3-3)	0.007

IHF Qol: Iranian questionnaire to assess quality of life in patients with heart failure, SD: Standard deviation, IQR: Interquartile range.

### Safety of allopurinol

The drug was well tolerated by our study population and there were no reports of hepatotoxicity, agranulocytosis, and thrombocytopenia and allopurinol hypersensitivity syndrome. Only one patient developed skin rash who was excluded from study.

## Discussion

In present study we found high dose allopurinol (600 mg) is well tolerated by HFrEF patients who have normal uric acid level and may have favorable hemodynamic and neurohormonal effects as shown by clinical status, exercise capacity, laboratory and strain imaging findings.

Using allopurinol in cardiovascular diseases stems from discoveries about XO as well as uric acid (UA) and its prognostic role in chronic HF.^2-8,10-13,16,16-19^

Although Exact–HF and OPT-CHF trials,^5,8^ the largest prospective, randomized, controlled trials testing the effect of XO inhibition on mortality and clinical outcomes in patients with HFrEF, failed to show beneficial effects of allopurinol on clinical status and outcomes of these patients, many experimental and clinical studies indicate allopurinol use reduces oxygen free radicals formation as well as oxidative damage and has favorable effects on myocardial energy metabolism, endothelial function, mechanoenergetic coupling, myocardial oxygen consumption, ventricular remodeling and clinical status in the setting of HF.^3,4,6-12^ Also it has been shown a trend toward decreased cardiovascular events and mortality with allopurinol use in many clinical studies.^8,18^ These remarkable therapeutic values of this drug in patients with HF seems to be independent from its uric acid lowering effects. However, the therapeutic regimens and the pre-treatment levels of uric acid still remain controversial and the doses and routes of administration to take favorable effect were different in clinical trials. Most of the studies in this regard are small studies which use different doses of allopurinol and it has been shown that the effect of allopurinol on endothelial function and UA lowering in HF is dose dependent and the drug is most useful with high doses or in patients with elevated UA level.^3,4,6,8,12,20^ For example, in the OPT-CHF trial, Hare et al reported that treatment with oxypurinol (the active metabolite of allopurinol) was associated with improved clinical status and survival only in moderate to severe HFrEF patients with hyperuricemia (UA>9.5 mg/dL).^8^ However, Baldus et al showed intravenous oxypurinol could have positive inotropic effect independent to endogenous release of catecholamines in patients with HFrEF who had normal UA level.^21^ In Rekhraj et al study the LV mass measured by cardiac magnetic resonance imaging decreased after high dose allopurinol (600 mg/d) for 9 months in patients with ischemic heart disease and normal UA level.^22^ Finally, George et al showed that forearm blood flow response to acetylcholine increased significantly with a dose of 600 mg/d allopurinol compared with both allopurinol 300 mg/d and placebo.^10^

It has been suggested that high-dose allopurinol improves endothelial function by reducing vascular oxidative stress and not by lowering uric acid. However, some scientists suggest that high dose allopurinol can profoundly negate the adverse effect of high urate and improve survival.^3,8,10,12^

In present study, we showed high dose allopurinol (600 mg/d) in short term can improve clinical and functional conditions, biomarkers and QOL of HFrEF patients with normal UA level who are still symptomatic despite guideline directed medical therapies.

We also showed favorable effect of allopurinol on deformation indices of left ventricle measured by strain imaging echocardiography. Only a few studies investigated the changes in echocardiographic parameters after treatment with allopurinol. Cingolani et al, in La Plata study assessed LV size and EF after treatment with oxypurinol and detected a tendency to decrease both LV end systolic and diastolic volume and significantly increase in LVEF following treatment with oxypurinol.^20^ For as far as we have researched and studied, the favorable effect of allopurinol on LV deformational indices has been shown in our study for the first time. It has been shown that the depression of myocardial contractility in patients with HF is not simply due to myocyte loss and disturbances in myocyte and vascular redox pathways involved but also the imbalance between nitric oxide and ROS such as hydrogen peroxide and superoxide has been considered as a central contributor to decreased myocardial function.^2,10,20,22,23^ Based on above pathophysiologic mechanisms and the reduction of the endogenous NO inhibitor asymmetric dimethylarginine (ADMA) by allopurinol, the improvement of myocardial mechanical efficiency can be explained.^2,23^

## Study limitations

Small sample size, being not randomized and lack of control group and long term follow up data are the main limitations of our study. The improved clinical and biochemical parameters may also be due to standard HF treatment regimen which highlights the need of randomization in future studies.

In conclusion, although our study is not a randomized clinical trial, the results have many similarities with previous evidences and shows allopurinol could be a cardiovascular drug to preserve cardiac function and alter the progression of HF in non-hyperuricemic patients. More investigations in this regard can shed the light on the exact role of XO inhibition in cardiovascular medicine.

## Ethical approval

The study was approved by research and ethics committee of Rajaie Cardiovascular Medical and Research Center and registered with the IRCT ID of IRCT2015112125171N1 in the Iranian registry of clinical trials website (http://www.irct.ir/). The written informed consent was obtained from all patients.

## Competing interests

None declared.

## Acknowledgments

This work was supported by the Research deputy of the Rajaie Cardiovascular Medical and Research Center, Iran University of Medical Sciences, Tehran, Iran. We would like to thank all patients participating in the study.
